# HROM: Learning High-Resolution Representation and Object-Aware Masks for Visual Object Tracking

**DOI:** 10.3390/s20174807

**Published:** 2020-08-26

**Authors:** Dawei Zhang, Zhonglong Zheng, Tianxiang Wang, Yiran He

**Affiliations:** Department of Computer Science, College of Mathematics and Computer Science, Zhejiang Normal University, No 688, Yingbin Road, Jinhua 321004, China; davidzhang@zjnu.edu.cn (D.Z.); 18329020065@163.com (T.W.); 201825200701@zjnu.edu.cn (Y.H.)

**Keywords:** Siamese network, high-resolution representation, multi-scale fusion, visual tracking, attention mechanisms, deformable convolution

## Abstract

Siamese network-based trackers consider tracking as features cross-correlation between the target template and the search region. Therefore, feature representation plays an important role for constructing a high-performance tracker. However, all existing Siamese networks extract the deep but low-resolution features of the entire patch, which is not robust enough to estimate the target bounding box accurately. In this work, to address this issue, we propose a novel high-resolution Siamese network, which connects the high-to-low resolution convolution streams in parallel as well as repeatedly exchanges the information across resolutions to maintain high-resolution representations. The resulting representation is semantically richer and spatially more precise by a simple yet effective multi-scale feature fusion strategy. Moreover, we exploit attention mechanisms to learn object-aware masks for adaptive feature refinement, and use deformable convolution to handle complex geometric transformations. This makes the target more discriminative against distractors and background. Without bells and whistles, extensive experiments on popular tracking benchmarks containing OTB100, UAV123, VOT2018 and LaSOT demonstrate that the proposed tracker achieves state-of-the-art performance and runs in real time, confirming its efficiency and effectiveness.

## 1. Introduction

Visual object tracking is an important and fundamental subject of computer vision. In this paper, we only consider the single object tracking problem. With the bounding box of an arbitrary object in the initial frame, a tracker is aimed to estimate the location and size of the target in subsequent frames of this sequence. It has diverse applications in numerous fields, such as video understanding, visual surveillance, augmented reality and human-computer interaction, etc. It is still a challenging task in some difficult scenes like illumination changes, deformation, background clutter, motion blur, occlusion, low-resolution, to name a few. Therefore, an accurate and robust real-time tracker is required to in real-life applications.

Recently, Convolutional Neural Networks (CNN) have demonstrated superior performance of feature extraction in various computer vision tasks including object classification [1] detection [2], and segmentation [3]. With the development of object tracking algorithms, deep features have also been developed to further pursue high performance. Compared to handcrafted features-based trackers like KCF [4], CACF [5] and BACF [6], CNN-based trackers [7,8,9,10] show greater potential and have significant advantages in accuracy and robustness. However, most Discriminative Correlation Filters (DCF)-based approaches [8,9] cannot meet the real-time requirement due to a time-consuming procedure. Another hidden danger is that feature extractors which originally designed for object recognition are not trained offline on tracking datasets. The key to high-performance trackers is to capture the object-aware features for a better discriminative representation of the specific target, which ensures well generalization for each domain.

To strike a balance between accuracy and speed, Siamese networks-based trackers [11,12,13,14] have developed into a promising solution. Due to the simple, neat and effective offline training strategy, these approaches which formulate tracking as a template matching problem have attracted wide attention in visual tracking community. Specifically, Siamese-based trackers [11,14] are aimed to learn a general similarity function via cross-correlation for an exemplar patch and the candidate patches, and perform tracking without any fine-tuning or online update process. To abandon original time-consuming multi-scale testing and guarantee tracking accuracy, SiamRPN [14] introduces a region proposal network [2] into the classic Siamese networks. DaSiamRPN [15] further improves the training strategy and enhances the discriminability of SiamRPN, obtaining state-of-the-art results on VOT challenges. To exploit more effective feature extractors, SiamRPN++ [16] successfully apply the modern networks [1,17] as backbone network to replace the shallow AlextNet [18]. These indicate that the performance of Siamese-based methods is highly reliant on the feature representation.

Despite these great progress, we observe that all Siamese-based trackers have built their backbone networks formed by concatenating feature maps from high to low resolution (e.g., AlexNet [18], MobileNet [17], ResNet [1]). These deep but low-resolution features of the entire image are not robust enough to estimate the object bounding box accurately. In addition, there are still some limitations for Siamese network-based tracking methods. First, the deep features offline learned sometimes cannot adapt well to the specific object in various videos. It is time-consuming to perform online learning or fine-tuning. Generally speaking, a certain range of background information is beneficial for tracking, but the context contains distracting objects could affect the quality of response maps. Second, the CNN features [19,20] is not invariant to large deformations, such as scale variations, rotation and occlusion. Therefore, Siamese-based trackers cannot handle well such complex geometric transformations.

In this paper, we propose a novel Siamese network with High-resolution Representation and Object-aware Masks (HROM) for visual object tracking to improve the discrimination and generalization capability of Siamese-based trackers. Specifically, we exploit the recent more advanced HRNet [21] for maintaining high-resolution representations and repeatedly fusing multi-scale features, which is essential for the position-sensitive tracking problem. Like SiamRPN++ [16], we use the same sampling strategy to overcome the spatial invariance restriction of Siamese-based trackers. As a result, we successfully train the framework of SiamRPN [14] using the modified HRNet containing four branches in parallel as backbone network and achieve significant improvement. Benefiting from the architecture, we develop a simple yet effective fusion strategy for multi-resolution features aggregation, which is helpful to improve the tracking accuracy. To the best of our knowledge, this work is the first high-resolution Siamese networks for visual tracking. Moreover, the proposed approach exploits the multi-level fused features at a high level to learn object-aware masks including channel and spatial dimensions, which achieves better discrimination ability. To guarantee tracking efficiency, all these learning procedures are conducted during the offline training phase. Extensive evaluations on several popular tracking benchmarks validate the effectiveness of the proposed tracker.

The main contributions of this work can be summarized as:

- We propose a novel high-resolution Siamese network for visual tracking. The architecture inherits the merits from ResNet [1] (residual skip connection) and HRNet [22] (connecting high-to-low resolution in parallel, exchanging the information across resolutions).
- We integrate the high-resolution representation and multi-level features aggregation structure with Siamese region proposal networks for visual tracking, which is helpful to predict the similarity map from features fused with different resolutions at multiple levels.
- We develop two types of attention mechanisms to learn object-aware masks for adaptive feature refinement, and exploit deformable convolution to handle large geometric transformations. The resulting features are robust to appearance changes of the object itself.
- Extensive experimental results, on several popular tracking benchmarks containing OTB-2015, LaSOT, VOT-2018, and UAV123, demonstrate that the proposed tracking algorithm achieves state-of-the-art results while performing at a real-time speed.

The rest of this article is organized as follows. Section 2 reviews object tracking, high-resolution representation and object-aware features briefly. Section 3 proposes a novel approach to learn high-resolution representation and object-aware masks for Siamese tracking. Section 4 conducts extensive comparative experiments and ablation analysis. Finally, Section 5 concludes this work.

## 2. Related Work

In this section, the related works about deep features-based tracking, Siamese networks-based trackers, high-resolution representation and object-aware features are reviewed briefly.

### 2.1. Deep Features-Based Tracking

With the development of deep learning technologies, CNNs provide an effective solution to learn powerful deep representations. Recently, deep features have been widely integrated into various tracking frameworks for better performance. For example, HCF [23] and DeepSRDCF [24] take full use of deep features from multiple layers of the pretrained VGG [25], and combine them with correlation filters. Furthermore, C-COT [26], ECO [8] and UPDT [9] use the continuous convolution operators to integrate feature maps with shallow and deep resolutions for higher accuracy. From another perspective, visual tracking can be regarded as a classification problem. CNN-SVM [27] uses the CNN features and a traditional SVM classifier to perform classification, while MDNet [7] employs an end-to-end pretrained CNN model to conduct online fine-tuning for domain-specific classification. Despite the excellent tracking accuracy in OTB-100 [28] and VOT-2015 [29], MDNet only runs 1 fps due to the particle filter sampling strategy. Subsequently, large of works [30,31,32] exploit reinforcement learning-based search strategy and an improved RoIAlign technique to speed up MDNet.

These approaches with online learning or update can adapt various appearance changes of the specific object, but they may tend to over-fitting and lose the target in case of occlusion or background clutter. More unfortunately, due to extra computational cost, online model update is so time-consuming that it is a bit difficult to meet a real-time requirement.

### 2.2. Siamese Network-Based Tracking

In recent years, Siamese networks-based trackers have shown great potential due to a good balance between accuracy and efficiency. As an early work, Siamese network consisting of two parallel branches is used to learn similarity metric for face verification [33]. To some extent, visual tracking can also be converted as a similarity learning task. For instance, SINT [34], a pioneering work, formulated tracking as a verification problem and exploited a Siamese network to learn object matching. Furthermore, GOTURN [12] exploited the concatenated features from consecutive frames to perform fast tracking by regression, while Bertinetto et al. proposed SiameseFC [11] to learn the similarity by performing efficient cross-correlation. Both can perform far beyond real-time online tracking without extra fine-tuning. Owing to the characteristics (neat, simple and efficient) in SiamFC, there are numerous follow-up improvements [13,35,36,37]. RASNet [13] explored a residual attention Siamese network to adapt the offline learned features representation to the tracked target, while SiamRPN [14] introduced a region proposal network into Siamese networks to simultaneously perform classification and regression for high-performance tracking. DaSiamRPN [15] obtains significant improvement by more effective offline training with large-scale image pairs. Recently, SiamRPN++ [16] exploit successfully ResNet by a simple yet effective sample strategy for Siamese-based tracking, which further improves the performance due to more powerful representation. Meanwhile, DiMP [38] proposed a discriminative model prediction architecture by fully exploiting background information and provided an effective optimization strategy to update the model, which ensures rapid convergence.

### 2.3. High-Resolution Representation

Feature representation plays a significant role in numerous computer vision tasks including image classification [1,18,25], object detection [2,39], segmentation [3]. Deep feature is beneficial to extract rich semantic information, while high-resolution representation is significant for position-sensitive vision problems, such as object detection, human pose estimation and semantic segmentation. Most approaches [40,41,42,43] use U-Net or encoder-decoder to recover the high-resolution representations from the low-resolution for pixel-level segmentation, while feature pyramids [44,45] are exploited to perform up-sample process for object detection. Recently, HRNet [21] is proposed to maintain high-resolution representations through connecting multi-resolution streams in parallel and conducting repeated multi-scale fusions, which shows the superiority in large range of visual applications.

In fact, visual tracking also requires high-resolution representation for more accurate location. We observe that all trackers extract the deep but low-resolution features of the entire image by CNNs, such as AlexNet [18], VGG [25], MobileNet [17], ResNet [1]. This is not robust enough to estimate the target bounding box accurately. In this work, we first attempt to exploit high-resolution representation for visual tracking, which is semantically richer and spatially more precise.

### 2.4. Object-Aware Features

Features extracted in most Siamese-based trackers can only distinguish the tracked object from the non-semantic backgrounds. When the background is chaotic or similar distractors occur, trackers are prone to drift due to unreliable similarity maps. Therefore, the object-aware features are essential for high-performance trackers. To this end, some works propose to learn a spatial attention mask that highlights the foreground area. CSRDCF [46] exploits a spatial reliability mask to constrain correlation filters, while SA-Siam [47] develops a two-fold Siamese network and uses a channel-wise attention module to represent the target. More comprehensively, RASNet [13] explores different types of attention mechanisms for only template branch to adapt the features representation to the specific tracking object. Recent TADT [48] develops a ranking loss and a regression loss to learn target-aware deep features for online tracking. In contrast to these methods, this work learns attention-guided spatial and channel masks for template and search branches to highlight the importance of object-aware features. Unlike TADT, our learning process is performed on the offline training stage.

## 3. The Proposed Tracking Framework

**Figure 1:**
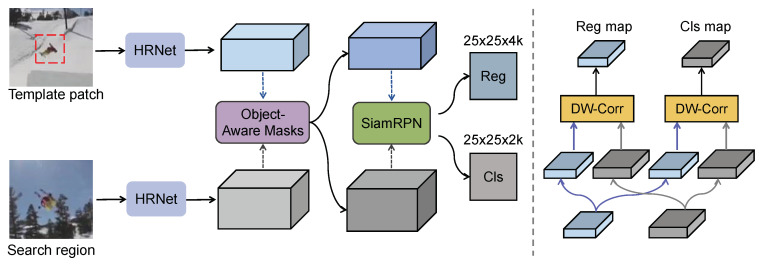
The overall tracking framework of the proposed HROM. The (**left**) figure illustrates our main structure, while the (**right**) one shows the RPN head in detail.

The overall framework of the proposed HROM is shown in Figure 1. Compared to the existing Siamese-based trackers, we integrate high-resolution representation into Siamese networks to extract richer multi-resolution features. We propose a simple yet effective structure of feature fusion at multiple levels for better localization. Moreover, different kinds of attention mechanisms are used to learn object-aware masks, which can adaptively highlight the target-aware features for discrimination.

### 3.1. Siamese Networks for Tracking

Siamese-based tracking algorithms [11,14,16] formulate tracking as a similarity learning problem, and use deep models to learn the similarity map by cross-correlation. The Siamese structure takes image pairs as input of two branches, where one branch extracts the deep features of an exemplar z, and another one severs for the candidate region x. Specifically, the image patch z usually indicates the object of interest given in the first frame of a sequence, while x with a larger size represents the search area in subsequent frames. Therefore, the similarity map between them can be measured in the learned embedding space θ(·):

(1) f(z,x)=θ(z)∗θ(x)+b

where *b* denotes the bias term, ∗ denotes the cross-correlation. Equation (1) equals to executing a complete matching of the target z over the search region x. To achieve accurate bounding box, SiamRPN [14] integrated RPN into Siamese networks, and exploited up-channel cross-correlation to perform classification and regression separately. Furthermore, SiamRPN++ [16] proposed an effective spatial-aware sampling scheme to alleviate the limitation of strict translation invariance caused by the padding operation, and unveiled the power of deep network like ResNet-50 to pursuit higher performance. In addition, SiamRPN++ [16] developed a depth-wise cross-correlation layer, which stabilizes the training process due to few and balanced parameters. Similarly, we apply the spatial-aware sampling strategy and depth-wise separable cross-correlation into our approach.

In contrast to SiamRPN++, we develop high-resolution networks to learn both fine-grained low-level representation and high-level semantic information in a mutual manner. SiamRPN++ performs the cross-correlation over multiple-scale features and aggregates the response maps by weighted sum. In this paper, we provide another simple yet effective scheme to aggregate multi-layer features by exploiting the recent more advanced HRNet. Moreover, we exploit different attention modules to learn the object-aware masks that enhance discrimination learning of the tracked target, which improves the target discriminability against complex backgrounds or distractors. A well-known fact is that the standard CNN features is not invariant to large deformations. To this end, we further attempt to exploit deformable convolution to handle complex geometric transformations. The resulting features are robust to appearance changes. In conclusion, we provide several effective and efficient offline training solutions for high-performance tracking on both accuracy and robustness.

### 3.2. High-Resolution Networks

**Figure 2:**
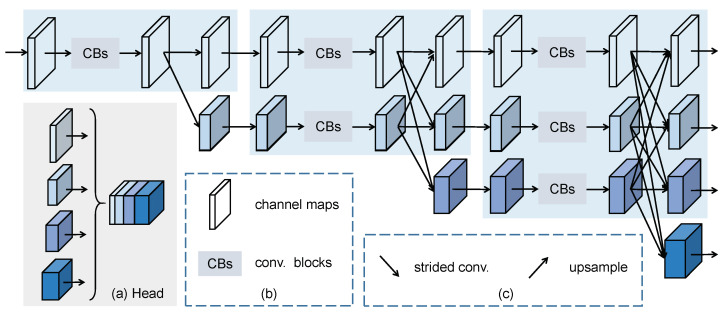
Illustration of the main structure in our HRNet. The 1st stage (light blue box) only has the high-resolution branch, while the 2nd (3rd) stage contains two-resolution (three-resolution) blocks.

Inspired by High-Resolution Network [22], we develop a carefully modified HRNet containing three stages as the shared backbone network, which can be end-to-end trained. The input is first fed into a stem consisting of two 3×3 convolutions with stride 2 for resolution reduction, and subsequently transmitted the main body that includes parallel multi-branch convolutions with different resolutions. The main structure is illustrated in Figure 2, while the network architecture is shown in Table 1.

Following the design of HRNet [22], we gradually append high-to low-resolution streams, forming the new stage consisting of the previous resolution and an extra lower one, and connect the multi-resolution branches in parallel. The advantage is that the resulting representation is more precise spatial location and richer semantic information. To ensuring efficiency, we remove the last stage of the original HRNet and only retain the first three stages as our backbone network. Table 1 shows that every stage is composed of modularized blocks, repeated 1, 1 and 4 times, respectively for the three stages. As shown in Figure 2, every branch comprised of four residual blocks and one multi-resolution fusion unit, corresponds to a different resolution. The fusion module is aimed to exchange the information across multi-resolution features by a transform function $T_{xr}$ (*x*, *r* are the branch index of input and output respectively), which is beneficial for the position-sensitive tracking problem. Taking the third stage of the second branch as an instance, the fused representation $R_{2}^{o}$ can be expressed as Equation (2) with the given three representations $\left\{R_{r}^{i},r=1,2,3\right\}$:

(2) $$R_{2}^{o}=T_{12}\left(R_{1}^{i}\right)+T_{22}\left(R_{2}^{i}\right)+T_{32}\left(R_{3}^{i}\right)$$

where $R_{r}^{o}$ is the output representation of the r-index branch, and $R_{r}^{i}$ means the input representation of the r-index branch. About the transform function, $T_{xr}\left(R\right)=R$ if x=r. If x>r, $T_{xr}\left(R\right)$ represents bilinear up-sampling followed by a 1×1 convolution for channel alignment. If x<r, $T_{xr}\left(R\right)$ down-samples the representation by a 3×3 convolution with stride 2.

The original HRNet [22] is not suitable for Siamese networks due to a large stride with 32. To be consistent with [14,16], we decrease the stride of the last stage from 16 to 8 by altering the residual blocks to keep a unified spatial stride. Please note that we expand its receptive field by exploiting dilated convolutions, which is also helpful for improving the representability of features with different scales.

Most existing methods [11,14] usually exploit the local features of the last layer. Even with the deep features, a single layer is not enough to infer precise classification and localization. Intuitively, rich representation that aggregates features from fine to coarse resolutions, from small to large scales, and from low to high levels is required for vision tasks [9,44,49]. Generally, feature from shallow layer mainly includes fine-grained low-level information such as shape and color, is essential for regression, while feature from deep layer captures rich high-level semantic information which is beneficial for discriminative classification. Based on this consideration, SiamRPN++ [16] performs the cross-correlation over multiple-layer features and integrates them by a weight layer.

In this work, we exploit the inherent advantage of the recent more advanced HRNet that maintains high-resolution representations and repeatedly fuses multi-scale features, and especially design a representation head to aggregate multi-level features for lightweight Siamese-based tracking. As illustrated in Figure 2a, we exploit the three representations of the third stage to obtain the features with 32 times receptive field through a transform fusion unit and dilated convolution layers. With the multi-scale representations, we first down-sample the high-resolution feature of the first branch through a 3×3 convolution to keep the same spatial resolution as others. Therefore, the multi-level features can be achieved by concatenating the four output representations. Finally, an extra 1×1 convolution layer is followed to mix the multi-level representations and reduce the channel to 256.

(3) $$R\left(X\right)={\mathrm{Conv}}^{1\times 1}\left(\mathrm{Cat}\left[{\mathrm{Conv}}^{3\times 3}\left(R_{1}^{o}\left(X\right)\right),R_{2}^{o}\left(X\right),R_{3}^{o}\left(X\right),R_{4}^{o}\left(X\right)\right]\right)$$

To alleviate a calculation burden of the cross-correlation layer, we cut out the center 8×8 spatial region [16] in 16×16 feature maps as the template representation.

### 3.3. Learning Object-Aware Masks

**Figure 3:**
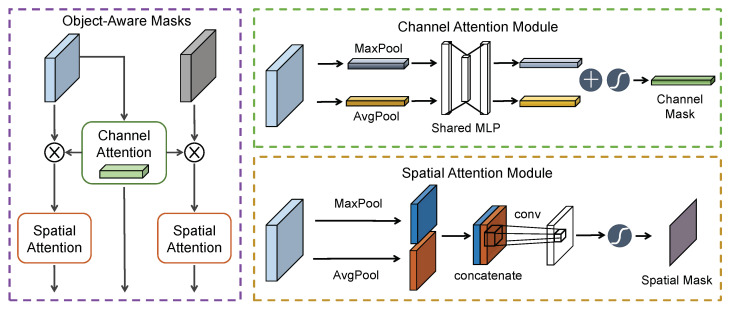
Illustration of the proposed object-aware mask module. The (**left**) figure illustrates the entire structure, while the (**right**) one shows each attention sub-module in detail.

Generally speaking, deep features captured by CNNs have strong representation ability, which achieves significant success in object recognition. Since visual tracking is aimed to track an arbitrary object of interest, generic representation is not effective to model the specific target for distinguishing it from the complex background. Therefore, under certain challenging factors, especially similar objects, background clutter, performance cannot be guaranteed. To this end, RASNet [13] proposes a residual attentional Siamese network for adapting the model to online tracking, while SA-Siam [47] exploits a channel attention module to capture channel-wise relationships. However, they only serve for the template branch, which might limit its discriminability due to ignoring the information in search branch. To address this issue, as shown in Figure 3, we develop two types of attention mechanisms to learn object-aware masks along two separate dimensions (channel and spatial) for both template and search branches. Benefiting from this effective and efficient solution, our approach obtains a certain performance gain with little extra computation and memory.

#### 3.3.1. Cross-Branch Channel Attention

Different channels often correspond to different kinds of visual pattern. In [16], as an interesting phenomenon, objects with the same category have high response on the same channels. Each channel represents certain semantic information. Therefore, a channel-wise attention mechanism is considered to be the procedure of allocating semantic attributes for different categories. Due to the pure target information in template patch, we develop a cross-branch channel attention module exploiting the relationship between channels in the template branch to learn a shared channel-wise mask for two branches. Please note that it is of great importance for the search branch to learn the target information, which contributes to a discriminative representation for identifying the target more accurately.

In more detail, as depicted in Figure 3, we first use both Global Maximum Pooling (GMP) and Global Average Pooling (GAP) to squeeze the spatial information of the feature maps, generating two context descriptors. It is helpful to improve representation compared to using each independently, because GMP reflects the salient target feature while GAP reflects the overall information of the target. Subsequently, both descriptors are fed into a shared Multi-Layer Perceptron (MLP) to produce the corresponding output feature vectors. We merge them using an element-wise summation operation followed by a sigmoid activation. In short, the channel mask can be computed as:

(4) $$M_{c}\left(X\right)=\sigma \left(\mathrm{MLP}\left[\mathrm{GAP}\left(X\right)\right]+\mathrm{MLP}\left[\mathrm{GMP}\left(X\right)\right]\right)$$

where σ means the sigmoid function, MLP consists of a fully connected layer for dimension reduction, a ReLU activation function as well as a fully connected layer for restoring the number of channels.

#### 3.3.2. Separable-Branch Spatial Attention

Intuitively, spatial attention is aimed to focus on where an important unit is, which is complementary with channel attention. Due to the different sizes of the two branches, we develop two separable-branch spatial attention modules. Therefore, the spatial attention is subsequently exploited to further suppress background interference or unimportant areas. To capture the spatial mask, we first use GAP and GMP along the channel axis to aggregate channel information, generating two feature descriptors, which are concatenated to preserve important information. Then, we exploit a 3×3 convolution layer to encode a spatial attention map, which can be expressed as:

(5) $$M_{s}\left(X\right)=\sigma \left({\mathrm{Conv}}^{3\times 3}\left(\mathrm{Cat}\left[\mathrm{GAP}\left(X\right),\mathrm{GMP}\left(X\right)\right]\right)\right)$$

To guarantee online tracking efficiency, the learning process of these modules is completed in the offline phase. Channel and spatial attention mechanisms can learn ‘what’ and ‘where’ to emphasize or restrain in the channel and spatial axes, respectively. Therefore, the attention maps can be multiplied to the representations generated by the backbone network for capturing adaptive object-aware features, which is beneficial to adapt the offline learned representation to an arbitrary object during tracking. Benefiting from the detachment of feature representation and discrimination learning, the proposed approach alleviates the over-fitting problem to some extent and improves the discriminative ability.

### 3.4. Deformable Asymmetric Region Proposals

During online tracking, the targets usually have large appearance changes, such as deformation, scale variation, rotation and partial occlusion. Intuitively, these geometric transformations break the symmetricity of feature matching, so an asymmetric design may help overcome this problem.

Based on the SiamRPN block, we introduce an extra 3×3 deformable convolution [50] to encode the content of target in search region. This is because the search branch is changing over time while the template branch remains fixed. Unlike the standard convolution, deformable convolution can sample at variable locations rather than a fixed position. Different locations in each convolution kernel are associated with different offsets, which is suitable for visual tracking.

(6) $$\begin{array}{cc} O_{cls}\left(x,z\right) & ={\left[\mathrm{DeConv}\left(M_{c}\left(x\right)\cdot M_{s}\left(x\right)\cdot R\left(x\right)\right)\right]}_{cls}☆{\left[M_{c}\left(x\right)\cdot M_{s}\left(z\right)\cdot R\left(z\right)\right]}_{cls}\\ O_{reg}\left(x,z\right) & ={\left[\mathrm{DeConv}\left(M_{c}\left(x\right)\cdot M_{s}\left(x\right)\cdot R\left(x\right)\right)\right]}_{reg}☆{\left[M_{c}\left(x\right)\cdot M_{s}\left(z\right)\cdot R\left(z\right)\right]}_{reg} \end{array}$$

where DeConv denotes a 3×3 deformable convolution, $M_{c}$ and $M_{s}$ denote the learned channel and spatial masks, and R is the representation generated by our backbone. All of them are beneficial to enhancing the discriminative representation of targets and performing classification and regression.

## 4. Experiments

In this section, we first provide the details of implementation. Then, ablation study is presented to analyze the effects of each component in the proposed tracker. Furthermore, the experimental results of HROM are compared on several popular tracking benchmarks including OTB2015 [28], UAV123 [51], VOT2018 [52] and LaSOT [53] with the state-of-the-art tracking algorithms.

### 4.1. Implementation Details

In the experiment, the training and inference settings are roughly similar to SiamRPN++ [16]. For consistent comparison, we crop and resize the template patch and search region of 127×127 and 255×255 respectively for both training and testing. The backbone network, the modified HRNet [22], is pretrained on ImageNet [54], which is an effective initialization for visual tracking. Like [16], we further retrain or fine-tune the whole model on the training sets of ImageNet VID [54], ImageNet DET, COCO [55], and YouTube-BB [56]. This is essential to learn a generic similarity measurement for Siamese-based tracking. In contrast to layer-wise cross-correlation aggregation, we only perform depth-wise cross-correlation of the fused multi-level features once. As a result, the produced response maps are adopted to perform proposal classification and box regression efficiently.

During training, HROM is trained with an optimization method of stochastic gradient descent and a batch size of 12 for 20 epochs. Specifically, for the first 10 epochs, we exploit the warm-up strategy with a fixed learning rate $10^{-3}$ to train the object-aware module and RPN branch. For the last 10 epochs, the whole model is trained with a learning rate decayed exponentially from $10^{-3}$ to $10^{-4}$ in an end-to-end mode. By following SiamRPN [14], we set anchor boxes with 5 aspect ratios, [0.33,0.5,1,2,3]. An anchor box is labeled as positive when IoU >0.6, and as negative when IoU <0.3. The loss is a summation of classification loss and standard smooth L1 loss for regression.

In the inference phase, the template patch is only computed once in the first frame, and then is continuously matched with subsequent search regions. To guarantee a stable and smooth tracking, we apply cosine window penalty, scale change penalty and linear interpolation to determine the final tracking results. The proposed HROM is implemented in Python with PyTorch 0.4.1 on a workstation with Intel(R) Xeon(R) CPU E5-2683 v4 @2.10 GHz and a NVIDIA GeForce GTX 1080 Ti GPU.

### 4.2. Ablation Analysis

In this subsection, we analyze the influence of each component in our approach. Ablation study on OTB-2015 [28] and VOT-2018 [52] datasets is conducted to show the improvement.

**Figure 4:**
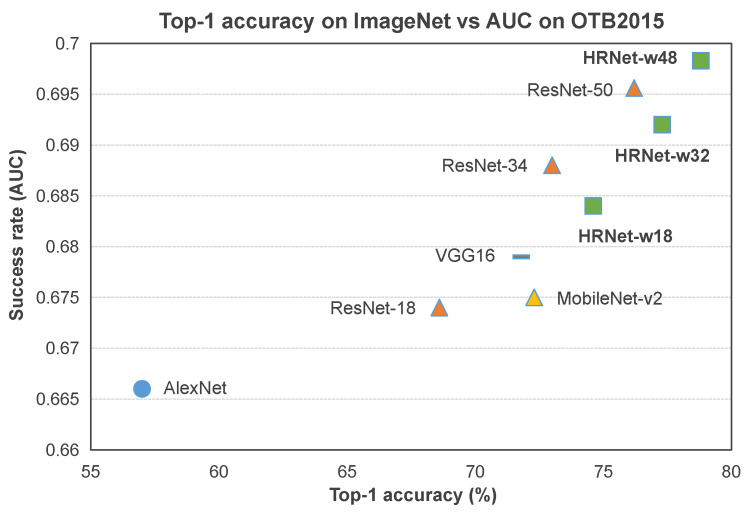
The Top-1 accuracy on ImageNet vs. the Area-Under-Curve (AUC) score on OTB-2015 [28].

**Backbone Architecture.** First, to evaluate the effect of high-resolution networks, we compare different backbone networks for Siamese-based tracking. We adopt SiamRPN++ [16] as the baseline. Figure 4 reports the performance of using AlexNet, VGG-16, ResNet-18, ResNet-34, ResNet-50, MobilNet-v2, HRNet-w18, HRNet-w32 and HRNet-w48 in terms of the top-1 accuracy on ImageNet and Area-Under-Curve (AUC) of success plot on OTB-2015. Benefiting from the high-resolution representation, our HRNets obtain relatively better performance. In particular, compared to the recent ResNet-50, HRNet-48 achieves the best AUC score with 0.698. This experiment shows that the backbone part of Siamese network is critical for tracking performance.

**Multi-Resolution Feature Aggregation.** We use different combination structures to analyze the impact of multi-level representations. To this end, we first use single representation from the last three branches on HRNet-18 for region proposal sub-network. From Table 2, We can observe that $R_{3}$ achieves a relatively good performance with EAO of 0.335, while $R_{2}$ and $R_{4}$ obtain 0.316 and 0.321 in EAO. Through concatenating two branches, a slight improvement can be gained. Furthermore, our tracker gets a 3.0% gain in EAO by aggregating $R_{2}$, $R_{3}$ and $R_{4}$, which proves the effectiveness of multi-level features fusion. Finally, we aggregate all representations from four branches and achieve the best performance with 0.353 in EAO on VOT-2018, which is higher than any single branch.

**Object-Aware Masks and Deformable Convolution.** In this study, we evaluate the influence of the proposed object-aware masks and deformable asymmetric region proposals. As shown in Table 3, with the deformable convolution, the EAO score improves from 0.353 to 0.362. Cross-branch channel attention and separable-branch spatial attention can improve the EAO by 1.9% and 1.2% respectively, which indicates that the learned cross-branch channel mask is a significant part to improve the performance, and even has a more important effect than the spatial mask. Moreover, the EAO score can be improved to 0.380 by Jointly exploiting both channel and spatial masks. The final model with object-aware masks and deformable convolution achieves a higher EAO of 0.387, improving both accuracy and robustness steadily, and exceeding the baseline by a large margin of 3.4%. Furthermore, by replacing the HRNet-w18 with HRNet-w32 and HRNet-w48, the EAO score can be improved to 0.415 and 0.436, respectively. It further proves that the high-resolution representation and object-aware masks are the primary factors to the performance improvement.

### 4.3. Comparison with the State-of-the-Arts

We further evaluate the proposed approach by compared to state-of-the-art tracking algorithms consisting of Siamese-based trackers and discriminative correlation-filter-based methods. Four datasets including VOT-2018, OTB-2015, UAV123 and LaSOT are employed to perform comparison.

#### 4.3.1. Evaluation on VOT-2018 Dataset

VOT-2018 [52] consisting of 60 public challenging video sequences, is one of the most popular benchmarks for testing and evaluating trackers. Generally speaking, Accuracy (A), Robustness (R) and Expected Average Overlap (EAO) are adopted to evaluate and rank all trackers. Following the official evaluation protocol of VOT-2018, We evaluate our HROM tracker on VOT-2018 dataset [52] in comparison with 13 state-of-the-art tracking methods including SiamFC [11], ECO [8], the top-7 trackers in VOT-2018 competition, ATOM [57], SiamRPN++ [16], SiamFC++ [58] and DiMP-50 [38].

As reported in Table 4, the proposed HROM-48 obtains the top accuracy and the second-rank EAO score. In particular, HROM-48 outperforms SiamRPN++ with a visible advantage in three metrics, which demonstrates the effectiveness of high-resolution and object-aware masks. Comparing the Top-1 tracker LADCF [10] in VOT-2018 challenge, our approach obtains an EAO gain of 4.7%. Moreover, compared with recent ATOM [57] and anchor-free tracker SiamFC++ [58], HROM-48 yields performance gains of 3.5% and 1.0% on EAO respectively. In addition, HROM-48 obtains a competitive EAO score with the recent leading tracker DiMP-50 [38]. About the robustness, our HROM also achieves a significant improvement, but still has a slight gap with DiMP-50 [38] which relies on the online learning.

**Figure 5:**
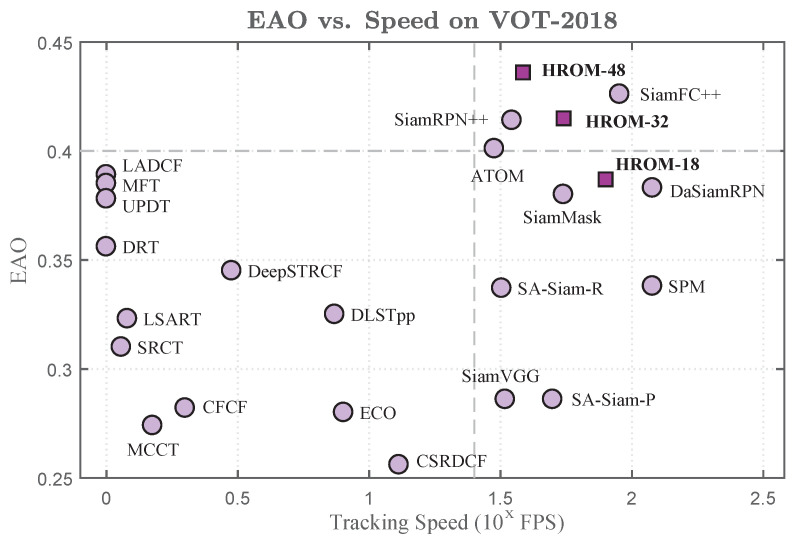
A comparison of the EAO scores and the speed with state-of-the-art tracking methods on VOT-2018 [52].

Furthermore, we compared the Expected Average Overlap (EAO) and the Frame-Per-Second (FPS) of state-of-the-art tracking methods on VOT-2018. Figure 5 shows the detailed comparisons. Please note that the tracking speed axis is in a log scale, and the gray dashed lines indicates the boundary. We can observe that HROM-48 achieves the best EAO score with a real-time tracking speed (40 fps). Two variants, which replace HRNet-w48 backbone with HRNet-w32 (HROM-32) and HRNet-w18 (HROM-18), respectively. HROM-32 obtains a competitive performance with SiamRPN++, and runs about 52 fps, while HROM-18 outperforms SiamMask [59] with a faster speed (78 fps).

#### 4.3.2. Evaluation on OTB Datasets

OTB-2015, a classic public tracking benchmark, is consisting of 100 sequences with 11 various challenging factors, such as Background Clutters (BC), Motion Blur (MB), Occlusion (OCC), Illumination Variation (IV), Deformation (DEF), Scale Variation (SV), Out-of-Plane Rotation (OPR), In-Plane Rotation (IPR), Fast Motion (FM), Out-of-View (OV) and Low Resolution (LR). Typically, One-Pass-Evaluation (OPE) is used to rank different tracking algorithms based on two criteria: center distance error at 20 pixels of precision plot as well as the AUC score of success plot.

**Figure 6:**
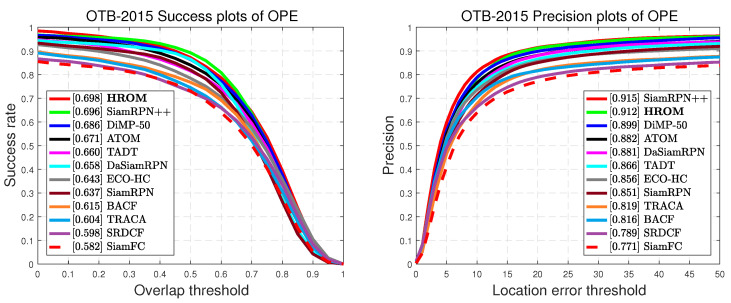
Success and precision plots on OTB-2015. The legend reports the area-under-the-curve scores of success plot and center-location-error at 20 pixel of precision plot for each tracker.

We compared HROM with 11 recent state-of-the-art tracking algorithms including DiMP-50 [38], SiamRPN++ [16], ATOM [57], TADT [48], DaSiamRPN [15], TRACA [60], ECO-HC [8], SiamRPN [14], BACF [6], SiamFC [11] and SRDCF [61]. Figure 6 reports both success and precision plots of these methods using OPE. Compared to SiamRPN++ [16], the proposed tracker obtains almost a similar performance with an AUC score of 0.698 and a distance precision rate of 0.912 at a more efficient design. Moreover, HROM outperforms recent leading tracker DiMP-50 [38] with relative gains of 1.2% and 1.3% for AUC and precision. HROM yields 3.8% gain in AUC of success plot than TADT. Both TADT [48] and our model explore the object-aware features, but the online learning of TADT hinders the generalization. In addition, compared to real-time correlation filter-based tracker ECO-HC [8], our method achieves 5.5% and 5.6% improvements in terms of success and precision, respectively. This shows that the proposed approach can perform effective and efficient online tracking.

**Figure 7:**
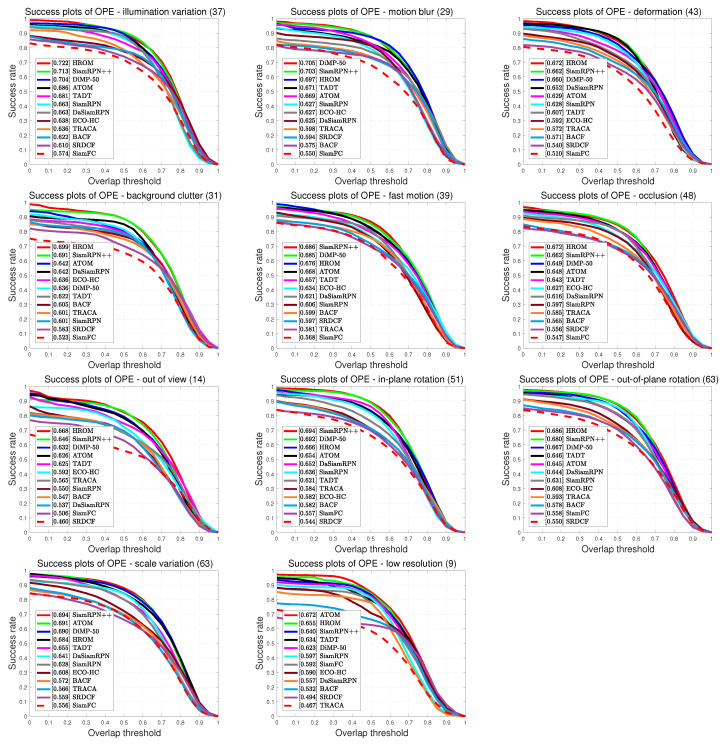
Success plots of different trackers with 11 attributes on the OTB-2015 benchmark.

**Figure 8:**
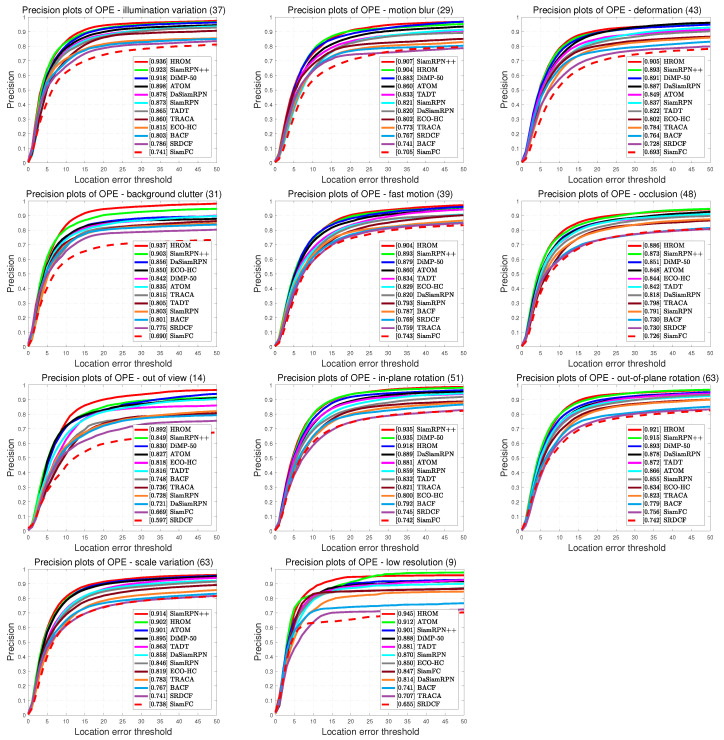
Precision plots of different trackers with 11 attributes on the OTB-2015 benchmark.

**Attribute-Based Performance Evaluation.** On the OTB benchmark, all sequences are annotated with 11 attributes for different challenging factors, namely Illumination Variation (IV), Motion Blur (MB), Deformation (DEF), Background Clutters (BC), Fast Motion (FM), Occlusion (OCC), Out-of-View (OV), Out-of-Plane Rotation (OPR), In-Plane Rotation (IPR), Scale Variation (SV) and Low Resolution (LR). To validate the effectiveness of high-resolution representations and object-aware features for visual tracking, we compare HROM with 11 trackers including DiMP-50 [38], SiamRPN++ [16], ATOM [57], TADT [48], DaSiamRPN [15], TRACA [60], ECO-HC [8], SiamRPN [14], BACF [6], SiamFC [11] and SRDCF [61] on OTB-2015. Figure 7 presents the overlap success plots of all challenging situations, and Figure 8 illustrates the distance precision plots for various tracking challenges.

As shown in Figure 7, our HROM outperforms other methods in 6 attributes: IV, DEF, BC, OCC, OV and OPR. Since deep features extracted from deeper networks are not sensitive to background clutter and illumination, SiamRPN++ performs not well. Benefiting from the high-resolution representation, our tracker achieves the top-rank performance and obtains 0.9% and 0.8% gains respectively than SiamRPN++. For LR attribute, HROM ranks 2nd with an AUC score of 0.655, outperforming SiamRPN++ with a relative gain of 1.5%. Moreover, our HROM works well in handling deformation, rotation and occlusion situations. This is mainly owed to asymmetric region proposals.

We also present distance precision plots for different challenges on OTB-2015. As illustrated in Figure 8. HROM achieves the top-rank precision in 8 attributes: IV, DEF, BC, FM, OCC, OV, OPR and LR. In particular, in BC and OV, the proposed method outperforms SiamRPN++ with obvious improvements of 3.4% and 4.3% respectively, which demonstrate the effectiveness of object-aware features. For SV, SiamRPN++ ranks the 1st with 0.914 due to complicated layer-wise feature aggregation, while our tracker ranks the second with 0.902. Furthermore, compared to SiamRPN++, HROM obtains a relative gain of 3.3% in LR due to the advantage of high-resolution resolution. In general, these results indicate that the proposed tracker can perform tracking effectively in most complex situations.

#### 4.3.3. Evaluation on UAV123 Dataset

UAV123 dataset contains 123 sequences captured by an UAV in a low-altitude aerial perspective. Like OTB, the success and precision plots also can be used to evaluate trackers. We compare HROM with other 9 state-of-the-art tracking methods containing SRDCF [61], SiamFC [11], ECO-HC [8], ECO [8], SiamRPN [14], MDNet [7], UPDT [9], DaSiamRPN [15], and SiamRPN++ [16]. Table 5 shows the comparing results in terms of precision and success. HROM obtains state-of-the-art performance (83.4% precision and 63.6% AUC), outperforming SiamRPN++ with relative gains of 2.7% and 2.3%. Compared with ATOM [57], HROM achieves a similar performance, which proves that our tracker is still effective to perform tracking in an aerial perspective due to the high-resolution representations.

#### 4.3.4. Evaluation on LaSOT Dataset

LaSOT [53] is a recent released more challenging benchmark for object tracking, and provides a high-quality and large-scale dense annotations with a total of 1400 sequences and 280 sequences for testing. Specifically, it includes 70 categories and each one contains 20 videos. Compared to the previous benchmarks, LaSOT aims to provide long-term sequences with an average of 2500 frames, which brings a great challenge to the trackers. Following the official protocol of LaSOT, we report precision, normalized precision plot, and success plots using one-pass-evaluation on the testing set.

**Figure 9:**
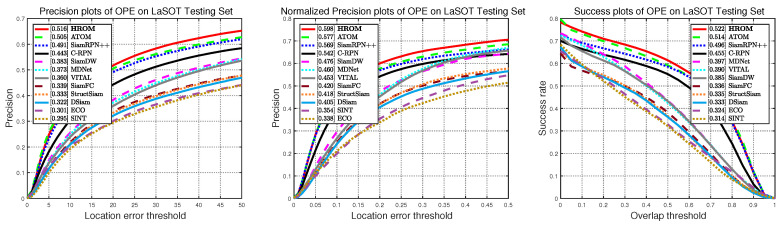
Precision, normalized precision and success plots on LaSOT.

To further evaluate the proposed tracker, we compare our HROM with 11 state-of-the-art methods consisting of SiamRPN++ [16], ATOM [57], C-RPN [62], SiamDW [63], VITAL [64], StructSiam [65], DSiam, ECO [8], MDNet [7], SiamFC [11] and SINT. As illustrated in Figure 9, the overall performance of HROM and other trackers are reported in detail on LaSOT testing set. Without bells and whistles, our HROM obtains the top-ranked performance with 51.6% precision, 59.8% normalized precision and 52.2% AUC. Compared with SiamRPN++ [16], HROM improves the performance by 2.5%, 2.9% and 2.6% relatively for three metrics, showing the impact of high-resolution representation. Compared with the best baseline MDNet [7] provided in the original paper, the proposed approach improves the ACU score by 12.5%. In addition, our HROM outperforms ATOM [57] with relative gains of 2.1% in normalized precision. These results prove a good generalization capability of our tracker.

#### 4.3.5. Qualitative Analysis

**Figure 10:**
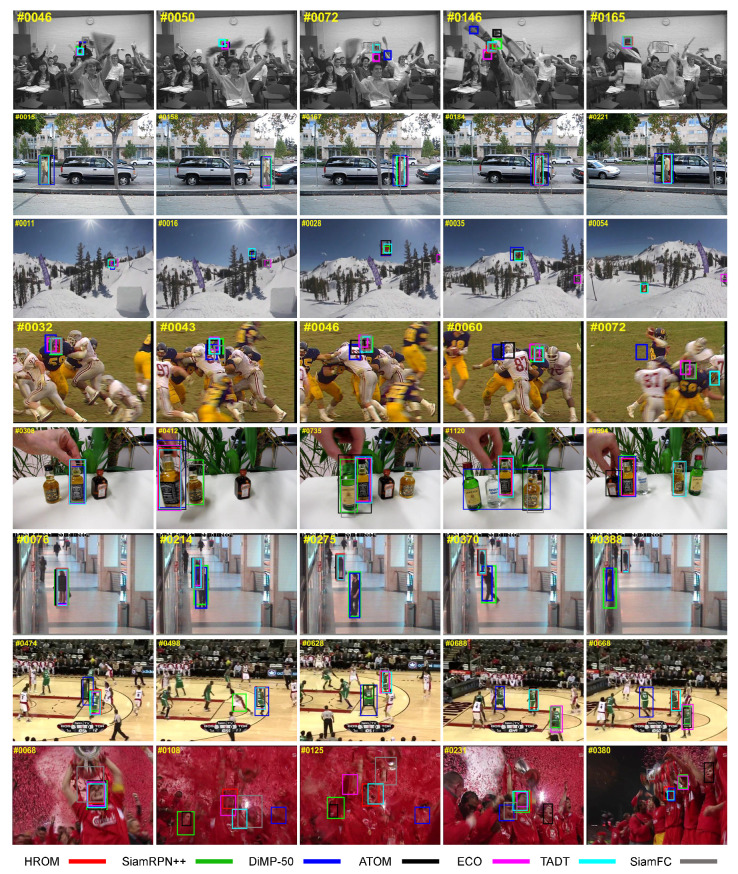
Qualitative evaluation of the proposed HROM and state-of-the-art tracking methods on the *Freeman4, David3, Skiing, Football1, Liquor, Walking2, Basketball* and *Soccer* sequences of OTB-2015.

As shown in Figure 10, we conduct the qualitative evaluation of 7 state-of-the-art trackers containing our HROM, SiamRPN++ [16], DiMP-50 [38], ATOM [57], ECO [8], TADT [48] and SiamFC [11] over several challenging sequences (from top to down: *Freeman4*, *David3*, *Skiing*, *Football1*, *Liquor*, *Walking2*, *Basketball* and *Soccer*) from OTB-2015 tracking benchmark [28].

*Freeman4*, *Skiing* and *Football* are instances of small object. Compared to other trackers, HROM provides more accurate bounding box due to high-resolution representation. For *Liquor* and *Basketball*, most trackers fail due to complex background or similar objects. Thanks to the object-aware features, our method and TADT perform better than others. *David3* and *Walking2* are examples of low resolution and partial occlusion, SiamRPN++ and DiMP-50 suffer a drift problem. Benefiting from high-resolution representation, our HROM tracks the target successfully. Due to illumination variation, motion blur and background clutter, *Soccer* is one of the most challenging sequences. All the trackers except HROM lose the tracked object occasionally due to different challenges. These shows that HROM is robust enough for most complex scenes including low resolution, background clutter and appearance variation of the object itself, etc.

## 5. Conclusions

In this work, we have presented a novel Siamese network-based framework with high-resolution representation and object-aware masks (HROM) for visual object tracking. Compared with previous deep Siamese networks, we connect the high- to low-resolution convolution streams in parallel and repeatedly exchanges the information across resolutions for high-resolution representation learning. To this end, we develop a simple yet effective module for multi-resolution feature aggregation, which assembles different levels of representations and is more suitable for position-sensitive tracking. In addition, different attention mechanisms are exploited to learn object-aware masks for enhancing the discrimination of targets. Furthermore, to handle large appearance variations, we explore asymmetric region proposals using deformable convolution for classification and regression. The resulting tracker greatly benefits from these schemes and achieves more robust and discriminative representation. Extensive experimental results demonstrate that the proposed tracker performs better than the state-of-the-arts while maintaining a real-time speed.

## Figures and Tables

**Table 1:** Architectures of our backbone network for Siamese-based trackers. There are three stages in total. In this table, each cell contains three components: the first [ ] is a residual unit, the second and third numbers means the repetition times of the residual units and the modualized blocks, respectively. For clarify, the fusion unit is not described. *C* in each residual unit is the number of channels.

Resolution	Stem	Stage 1	Stage 2	Stage 3
1/4	$\left[\begin{array}{c} 3\times 3,64\\ 3\times 3,64 \end{array}\right]\times 1$	$\left[\begin{array}{c} 1\times 1,64\\ 3\times 3,64\\ 1\times 1,256 \end{array}\right]\times 4\times 1$	$\left[\begin{array}{c} 3\times 3,C\\ 3\times 3,C \end{array}\right]\times 4\times 1$	$\left[\begin{array}{c} 3\times 3,C\\ 3\times 3,C \end{array}\right]\times 4\times 4$
1/8			$\left[\begin{array}{c} 3\times 3,2C\\ 3\times 3,2C \end{array}\right]\times 4\times 1$	$\left[\begin{array}{c} 3\times 3,2C\\ 3\times 3,2C \end{array}\right]\times 4\times 4$
1/8				$\left[\begin{array}{c} 3\times 3,4C\\ 3\times 3,4C \end{array}\right]\times 4\times 4$

**Table 2:** Analysis of the impact of different representation heads in HRNet-w18 on VOT-2018 dataset.

BackBone Networks	Rep Head	VOT-2018
HRNet-w18	$R_{2}$	0.316
HRNet-w18	$R_{3}$	0.335
HRNet-w18	$R_{4}$	0.321
HRNet-w18	$R_{2,3}$	0.338
HRNet-w18	$R_{2,4}$	0.325
HRNet-w18	$R_{3,4}$	0.344
HRNet-w18	$R_{2,3,4}$	0.349
HRNet-w18	$R_{1,2,3,4}$	**0.353**

**Table 3:** Ablation study of the proposed different modules on VOT2018 with EAO score. CM: Channel Mask, SM: Spatial Mask, DeCon: Deformable Convolution.

BackBone	CM	SM	DeConv	VOT-2018
HRNet-w18				0.353
HRNet-w18	✓			0.372
HRNet-w18		✓		0.365
HRNet-w18			✓	0.362
HRNet-w18	✓	✓		0.380
HRNet-w18	✓	✓	✓	0.387
HRNet-w32	✓	✓	✓	0.415
HRNet-w48	✓	✓	✓	**0.436**

**Table 4:** Comparison with 14 tracking methods on VOT-2018 benchmark. The red font and blue font represent the top-ranking and second-ranking performance, respectively.

Trackers	Accuracy ↑	Robustness ↓	EAO ↑
SiamFC	0.503	0.585	0.188
ECO	0.484	0.276	0.280
DeepSTRCF	0.523	0.215	0.345
DRT	0.519	0.201	0.356
RCO	0.507	0.155	0.376
UPDT	0.536	0.184	0.379
SiamRPN	0.588	0.276	0.383
MFT	0.505	0.140	0.385
LADCF	0.503	0.159	0.389
ATOM	0.590	0.204	0.401
SiamRPN++	0.600	0.234	0.414
SiamFC++	0.587	0.183	0.426
DiMP-50	0.596	0.153	0.440
**HROM-48**	0.606	0.173	0.436

**Table 5:** Comparisons on UAV-123. Red and blue fonts indicate the top-2 trackers.

Trackers	Precision (%)	Success (AUC) (%)
SRDCF	67.6	46.4
SiamFC	72.6	49.8
ECO-HC	72.5	50.6
ECO	74.1	52.5
SiamRPN	74.8	52.7
MDNet	77.2	52.8
UPDT	78.0	54.7
DaSiamRPN	79.6	58.6
SiamRPN++	80.7	61.3
ATOM	84.3	63.1
HROM	83.4	63.6
